# Extracellular vesicle-mediated intercellular and interorgan crosstalk of pancreatic islet in health and diabetes

**DOI:** 10.3389/fendo.2023.1170237

**Published:** 2023-05-25

**Authors:** Junlun Wei, Zhenghao Wang, Tingrui Han, Jiaoting Chen, Yiran Ou, Lan Wei, Xinyue Zhu, Ke Wang, Zhe Yan, Yuan-Ping Han, Xiaofeng Zheng

**Affiliations:** ^1^ Department of Endocrinology and Metabolism, Center for Diabetes and Metabolism Research, West China Hospital, Sichuan University, Chengdu, China; ^2^ The Rolf Luft Research Center for Diabetes and Endocrinology, Karolinska Institute, Stockholm, Sweden; ^3^ Department of Vascular Surgery, University Hospital of Chengdu University of Traditional Chinese Medicine, Chengdu, China; ^4^ The Center for Growth, Metabolism and Aging, The College of Life Sciences, Sichuan University, Chengdu, China

**Keywords:** diabetes mellitus, extracellular vesicles, pancreatic islet, organ crosstalk, therapeutic agent

## Abstract

Diabetes mellitus (DM) is a systemic metabolic disease with high mortality and morbidity. Extracellular vesicles (EVs) have emerged as a novel class of signaling molecules, biomarkers and therapeutic agents. EVs-mediated intercellular and interorgan crosstalk of pancreatic islets plays a crucial role in the regulation of insulin secretion of β-cells and insulin action in peripheral insulin target tissues, maintaining glucose homeostasis under physiological conditions, and it’s also involved in pathological changes including autoimmune response, insulin resistance and β-cell failure associated with DM. In addition, EVs may serve as biomarkers and therapeutic agents that respectively reflect the status and improve function and viability of pancreatic islets. In this review, we provide an overview of EVs, discuss EVs-mediated intercellular and interorgan crosstalk of pancreatic islet under physiological and diabetic conditions, and summarize the emerging applications of EVs in the diagnosis and treatment of DM. A better understanding of EVs-mediated intercellular and interorgan communication of pancreatic islets will broaden and enrich our knowledge of physiological homeostasis maintenance as well as the development, diagnosis and treatment of DM.

## 1 Introduction

Diabetes mellitus (DM) is a systemic metabolic disease characterized by hyperglycemia with high mortality and morbidity (1). The International Diabetes Federation estimates that 10.5% of people aged 20 to 79 years old are currently suffering with DM and the incidence will rise to 11.3% by 2030 and to 12.2% by 2045 (2). Long-term hyperglycemia brings a higher risk of neurovascular injury, which leads to a series of complications such as cardiovascular disease, diabetic neuropathy, diabetic kidney disease and diabetic retinopathy, in association with negative impacts on patients’ quality of life and heavy economic burdens (3). DM can be mainly classified into type 1 diabetes mellitus (T1DM) and type 2 diabetes mellitus (T2DM) based on the pathogenesis (4). Additionally, DM can be the secondary disease caused by Coxsackie-viral infection (5), cystic fibrosis (6) and pancreatic cancer (7). Although the etiologies of different types of DM are distinct, it is generally accepted that the progression of DM is highly associated with abnormal communications between cells, tissues and organs (8).

Extracellular vesicles (EVs) are a group of heterogenous, multi-functional lipid bilayer membrane vesicles secreted by a wide spectrum of cells into extracellular space (9). The characteristics of EVs are highly heterogenous and influenced by the parental cell types (10). EVs contain various biologically active molecules such as nucleic acids, proteins and lipids, where the bioactivity of their contents could be preserved by the membrane structure, making EVs as suitable carriers transmitting signals among cells (11). Over the last decade, EVs have garnered great interest for their critical roles in mediating intercellular and interorgan communications, which have been demonstrated to be involved in various physiological and pathological processes (12). Beside their intrinsic bioactive properties, EVs have also been confirmed with other advantages, such as abundant sources, low immunogenicity, biocompatibility, flexibility to modify, and ability to cross biological barriers (13), making EVs-based therapies as attractive strategies in the treatment of various diseases (14–16).

The pancreatic islet discovered by Paul Langerhans in 1869 consist of its own vasculature and five types of hormone-producing cells known as α-cells, β-cells, δ-cells, pp-cells and rare ϵ-cells (17). The hormones produced by different endocrine cells play a crucial role in controlling blood glucose level, making pancreatic islet an important mini-organ to maintain glucose homeostasis (18). Among different cell types, insulin-secreting β-cells occupy a decisive position given that loss and failure of β-cells are highly associated with the emergence and deterioration of DM (19). Present researches indicate that EVs-mediated crosstalk between pancreatic islets and extra-islet tissues plays key roles in maintaining glucose homeostasis under physiological conditions, whereas such crosstalk is strongly involved in the occurrence and development of DM under pathological conditions (8, 20). Under physiological conditions, pancreatic β-cells can lower blood glucose by secreting insulin to increase glucose uptake of peripheral tissues, while peripheral tissues may promote insulin secretion and growth of β-cells *via* EVs-mediated crosstalk (21, 22). On the other hand, EVs containing autoantigens derived from pancreatic islets may target autoimmune cells, causing severe autoimmune response in T1DM. Furthermore, peripheral tissues-derived EVs impose negative impacts on pancreatic islets, leading to dysfunction and death of islet cells in T2DM (23). EVs-mediated crosstalk between pancreatic islets and other tissues can also lead to the secondary diabetes after other diseases (6, 7). Notably, EVs have also been widely applied as biomarkers and therapeutic agents for DM. Lakhter et al. reported that up-regulated miR-21-5p cargo in β-cell derived EVs in response to inflammatory cytokines can serve as a potential biomarker of T1DM (24). Sun et al. showed that EVs secreted by mesenchymal stem cells (MSCs) can be used for the treatment of T2DM by promoting survival and insulin secretion of pancreatic β-cells (25).

This review aims to outline the current knowledge of EVs-mediated intercellular and interorgan crosstalk of pancreatic islet, focusing on its roles in regulating systemic metabolism, as well as its potential applications in the diagnosis and treatment of DM. We hope to provide a new and specific perspective for understanding certain physiological and pathological processes and give new thoughts to novel biomarkers discovery and anti-diabetic drug development.

## 2 Overview of EVs

EVs can be broadly divided into two major categories based on their characteristics of biogenesis: exosomes (30-100 nm) and microvesicles (MVs, 50-1000 nm) (11). Exosomes are formed as intraluminal vesicles (ILVs) *via* inward budding of endosomal membrane within the lumen of multivesicular endosomes (MVEs), which is released to extracellular space upon fusion of MVEs with plasma membrane (26). While, MVs are formed by outward blebbing and shedding from the plasma membrane (27). Apart from exosomes and MVs, increasing number of EV subpopulations including migrasomes (28), secretory amphisomes (29), exophers (30), apoptotic bodies (31) and endogenous retroviral-like particles (32) have also been reported. Roles of exosomes and MVs in intercellular or interorgan communication have been recently acknowledged (9, 33). These two main categories of EVs participate in communications between different cell types *via* two steps in a nutshell: generation and targeting to recipient cells.

## 3 Generation

### 3.1 Exosomes

The generation of exosomes can be generally categorized into three processes including biogenesis, transport and release. In the process of biogenesis, cargos fated for secretion are first targeted to endosomes, then followed by clustering, budding and fission to form ILVs *via* endosomal sorting complex required for transport (ESCRT)-dependent or ESCRT-independent pathway (34, 35). Many sorting mechanisms have been proven to be involved in exosomes biogenesis, while endosomal sorting machineries seem to be the main determinants (11). The biogenesis of ILVs is followed by transport of MVEs towards the plasma membrane. This process requires the association of MVEs with intracellular trafficking molecules, such as cytoskeleton, relevant molecular motors and molecular switches (36, 37). MVE-plasma membrane fusion mediated by SNARE proteins and synaptotagmin family members is the final step of exosomes generation (38), which triggers the release of ILVs into extracellular space.

### 3.2 MVs

MVs are usually generated at the protrusions of the plasma membrane, such as filipodia and microvili (39). The biogenesis of MVs requires several molecular rearrangements of the plasma membrane, participation of cytoskeletal elements and their regulators, which is tightly associated with metabolic changes of cells (40–42). Once formed, MVs is secreted to extracellular milieu *via* pinching off from the plasma membrane.

### 3.3 EV cargos

Cargos contained in EVs vary depending on the cell type, pathophysiological state and the extracellular environment (43). The cargo capacity of EVs can be influenced by a variety of factors, such as the size and abundance of the cargo molecule, the presence of specific transporters and the intrinsic ability of EVs to package and release (9). For example, studies have demonstrated that small non-coding RNAs, such as miRNAs, can be highly enriched in EVs, whereas larger RNAs, such as mRNAs, may be present in low quantities (44). As another example, some proteins on the surface of EVs, such as teraspanins and integrins, can interact with specific ligands on cargo molecules and facilitate their packaging into EVs (45). Additionally, RNA-binding proteins such as hnRNPA2B1 and YBX1 can also play a role in the selective sorting of RNA into EVs (46). **Table 1** lists the cargos packaged in EVs that participate in the pathophysiological process of DM.

**Table 1:** Type of EV cargos involved in the pathophysiology of DM.

Type of cargos	Examples
regulatory cargos	DNAs, mRNAs, tRNAs, miRNAs, IncRNAs, circRNAs, transcription factors, histones
inflammatory cargos	cytokines, chemokines
metabolic cargos	glucose transporters, lipid transporters, hormones and signaling molecules, metabolites
cellular cargos	MSC-derived, immunocyte-derived, endothelial cell-derived, adipocyte-derived

### 3.4 Targeting to recipient cells

EVs can be delivered to recipient cells to mediate intercellular communications in two steps: docking at the plasma membrane and transmitting signals to target cells. Binding of EVs to recipient cells is principally mediated by specific interactions between EV surface proteins and plasma membrane receptors of target cells (47). Upon binding, EVs can transmit signals by either activating the plasma membrane receptors or influencing various responses and processes after internalization (48). The release of molecules from EVs is regulated by various mechanisms, such as the fusion of EV membrane with b of the recipient cell or uptake by endocytosis (11). These processes involve specific proteins and lipids that mediate the interaction between EV membrane and recipient cell membrane, eventually leading to release of cargo molecules into the cytoplasm (49). For instance, the exosomal protein CD63 is involved in the fusion of exosomes with recipient cells while the lipid phosphatidylserine (PS) on the exosome membrane is recognized by the PS receptor on recipient cells, facilitating uptake of exosome cargos (50). In some cases, EVs can also be taken up by other mechanisms, such as micropinocytosis or phagocytosis (51). The internalized EVs will be mainly degraded by lysosome, which is influenced by specific compositions of EVs and specific structures at the plasma membrane of target cells (11, 52, 53).

## 4 EVs-mediated intercellular and interorgan crosstalk of pancreatic islet

### 4.1 Physiological status

Previous study has shown that β-cells secrete EVs containing higher levels of miR-223 upon high-glucose stimulation, which can subsequently upregulate GLUT4 expression in hepatocytes and skeletal muscle to facilitate glucose uptake (65). This phenomenon highlights the critical roles of EVs-mediated interorgan crosstalk in the maintenance of glucose homeostasis.

Adipose tissue is an endocrinal organ that involves in the regulation of immunity, insulin sensitivity, blood glucose and lipid metabolism, which is crucial for modulating internal environment homeostasis (66). Interestingly, adipocytes have been demonstrated to participate in the regulation of the physiological function of pancreatic islet cells through EVs-mediated crosstalk. EVs derived from healthy adipocytes can be delivered to recipient β-cells and induce the expression of transcription factors including Pdx1 and Nkx6.1, which can promote β-cell proliferation and insulin secretion, while prevent β-cell apoptosis (21).

Human islet amyloid polypeptide (hIAPP) is an important insulin co-secreted hormone (67), while hIAPP misfolding and aggregation may induce β-cells damage (68). Ribeiro et al. discovered that pancreatic islet-derived EVs can reduce hIAPP formation of pancreatic islet cells, suggesting a self-protection mechanism of pancreatic islets in physiological status (22). In a separate study, Shen et al. reported that β-cells-derived EVs can act upon recipient islet endothelial cells to promote the migration and tube formation through the action of cargo miR-127 (69).

Taken together, pancreatic islet cells can release EVs for communication with other tissues to achieve their biofunction. In turn, extra-islet tissues or pancreatic islet itself can also regulate pancreatic islet physiology *via* EV-mediated intercellular and interorgan crosstalk.

### 4.2 T1DM

**Figure 1:**
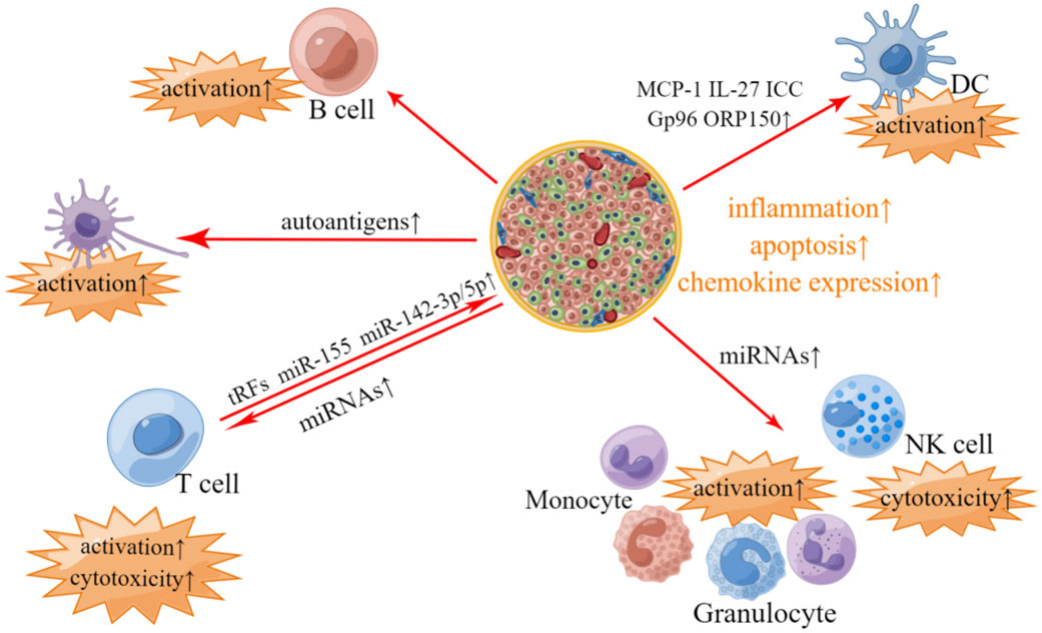
Involvement of islets- and immunocyte- derived EVs in T1DM-related pathological changes. In T1DM, pancreatic islet-derived EVs contain autoantigens, which can stimulate APC and promote T-cells and B-cells activation. Pancreatic islet-derived EVs can also modulate immune responses in an APC-independant manner, where differentially expressed miRNAs including miR-122-5p, miR-192-5p, miR185-5p, miR-195-3p, miR-455-5p, miR-375-3p and miR-129-5p in pancreatic islet-derived EVs may account for the activation of phagocytes (monocytes and granulocytes) and elevated cytotoxicity of T-cells and NK cells. EVs derived from pancreatic islets also contain other bioactive materials, such as MCP-1, IL-27, ICC, Gp96 and ORP150, which are associated with the activation of DC. Respectively, EVs derived from T-cells contain tRFs, miR-142-3p, miR-142-5p and miR-155, which may impose the deleterious impacts on pancreatic β-cells.

T1DM is known as autoimmune diabetes, characterized by immune cells infiltration, β-cell failure and insulin deficiency (70). The occurrence and deterioration of T1DM are proved to be closely associated with the crosstalk between pancreatic islet cells and immune cells (71). Pancreatic islets can promote the inflammation and autoimmune response by secreting EVs containing potent immunostimulatory materials. Respectively, immune cells derived EVs can induce damage and dysfunction of β-cells (72). Overall, EVs-mediated crosstalk between pancreatic islet cells and immune cells runs through the whole development of T1DM (**Figure 1**).

Collective evidence suggests that pancreatic islets derived EVs contain various autoantigens including GAD65, IA-2, ZnT8, Glut2, proinsulin, insulin, catabolites of insulin and newly discovered Gag antigen, which can stimulate antigen presenting cells (APC) and further activate T-cells and B-cells to trigger autoimmune response (73–77). In addition, EVs derived from pancreatic islets can also promote the activation of T-cells and B-cells in an APC-independent manner (78). A study by Tesovnik et al. reported that a great number of microRNAs (miRNAs) including miR-122-5p, miR-192-5p, miR-185-5p, miR-195-3p, miR-455-5p, miR-375-3p and miR-129-5p were differentially expressed in pancreatic islet-derived EVs in T1DM compared to that of healthy control, which can activate phagocytes *via* TLR7/8-mediated immune modulation and elevate the cytotoxicity of T-cells and NK cells (79). EVs derived from inflammatory islet cells also contain other bioactive materials including monocyte chemoattractant protein 1 (MCP-1), IL-27, immunostimulatory chaperones calreticulin, Gp96 and ORP150, which can activate dendritic cells and promote the release of pro-inflammatory cytokines (74, 80).

Respectively, T-cells can induce inflammation and apoptosis of pancreatic β-cells *via* tRNA-derived fragments (tRFs) contained in T-cell-derived EVs (81). In addition, T-cells derived EVs contain specific miRNAs such as miR-142-3p, miR-142-5p and miR-155, which can trigger chemokine expression and failure of pancreatic β cells (82).

### 4.3 T2DM

The process of T2DM involves two key mechanisms, insulin resistance (IR) and β-cell failure, accompanied with inflammatory response throughout the whole process (83). IR, a state in which insulin effector organs become less responsive to insulin, is highly correlated with obesity (84). During obesity, an increased mass of stored triglyceride in adipose tissue can induce IR in adipocytes through hypoxic response and inflammation (85). This results in enhanced lipolysis and increased release and circulating levels of free fatty acids (FFAs), which elicit lipotoxicity and aggravate IR in liver and skeletal muscle (86, 87). Upon the emergence of IR, current evidence strongly indicates that the mechanisms by which pancreatic islets compensate to cope with systemic IR are hyperplasia accompanied by excess insulin secretion and deposition of hIAPP in β-cells (88, 89). Meanwhile, chronic elevated FFAs and glucose caused by IR will induce β-cell dysfunction through endoplasmic reticulum stress (ER stress) and inflammation response (90, 91).

#### 4.3.1 From pancreatic islets to peripheral tissues: EVs serve as the initiator of IR

Previous research has reported that EVs derived from low-density lipoprotein (LDL) treated β-cells can induce insulin signal impairment by decreasing mTOR/p70S6Kα activation and lead to IR in hepatocytes (92). Hepatic IR has also been proven to be associated with up-regulation of miR-29s in EVs derived from free fatty acids (FFAs)-treated islets or down-regulation of miR-26a in EVs derived from islets of obese mice (93, 94). In addition, EVs derived from lncRNA Reg1cp mutated β-cells can transfer Mut-Reg1cp into hepatocytes, adipose tissue and skeletal muscle, and trigger peripheral IR by inhibiting AdipoR1 translation and adiponectin signaling (95). Systemic inflammation can further worsen IR of peripheral tissues in the pathogenesis of T2DM (96). Sun et al. showed that prediabetic pancreatic β-cells secrete EVs containing miR-29, which can promote the transformation of monocytes and macrophages to an inflammatory phenotype and improve their tissue residential ability, leading to the exacerbation of IR (97).

#### 4.3.2 From peripheral tissues to pancreatic islets: EVs serve as the initiator of β-cell compensation and failure

In the early stage of T2DM, β-cell compensation is one of the important mechanisms to delay the progression of disease (98). Accumulating evidences suggest that peripheral tissues can promote β-cell compensatory hyperplasia *via* EVs under prediabetic conditions. The study by Fu et al. reported that down-regulated miR-7218-5p in hepatocellular EVs derived from high-fat diet (HFD) induced obese mice can promote β-cell proliferation by targeting CD74 gene without affecting insulin secretion (99). Jalabert et al. demonstrated that up-regulated miR-16 in EVs derived from lipid-induced insulin-resistant muscles can promote β-cell proliferation by down-regulating Ptch1 of the hedgehog pathway (100). Additionally, miR-155 enriched EVs derived from adipose tissue macrophages can suppress Mafb expression in β-cells, leading to reduced insulin secretion and enhanced β-cell proliferation (101).

In the later stage of T2DM, EVs-mediated crosstalk between pancreatic islet and extra-islet tissues can lead to apoptosis and dysfunction of β-cells. Lipotoxic hepatocytes-derived EVs can augment immune cell infiltration in pancreas and subsequent β-cell failure (102). Inflamed adipose tissue can secrete EVs to induce β-cell death and dysfunction, while palmitate (PA)-induced IR skeletal muscle can also reduce the insulin secretion of β-cells *via* EVs-mediated crosstalk (21, 103). The intestinal flora of healthy people can maintain intestinal homeostasis by secreting metabolites, while the intestinal microorganism of obese people may induce inflammatory responses of β-cells in the context of T2DM (104, 105). The study by Gao et al. demonstrated that EVs derived from gut microbiota of obese subjects can pass through gut barrier and deliver microbial DNAs to pancreatic β-cells, resulting in islet inflammation and β-cell dysfunction (106). Qian et al. showed that EVs derived from islet-resident M1 macrophages of obese mice can transfer miR-212-5p into the adjacent pancreatic β-cells, leading to β-cell dysfunction by inhibiting SIRT2 (107).

#### 4.3.3 Self-regulation of pancreatic islets: EVs serve as the initiator of β-cell failure

Pancreatic islet cell-derived EVs is crucial in mediating autocrine and paracrine interactions within pancreatic islet, which may mediate negative self-regulation in the context of T2DM (108). EVs containing active procoagulant tissue factor derived from inflammatory β-cells can impose negative impacts on other β-cells and induce β‐cell dysfunction (109). Similarly, EVs containing miRNAs derived from inflammatory cytokine-treated β-cells can be transferred into the recipient β-cells and induce β-cell death (110).

**Figure 2:**
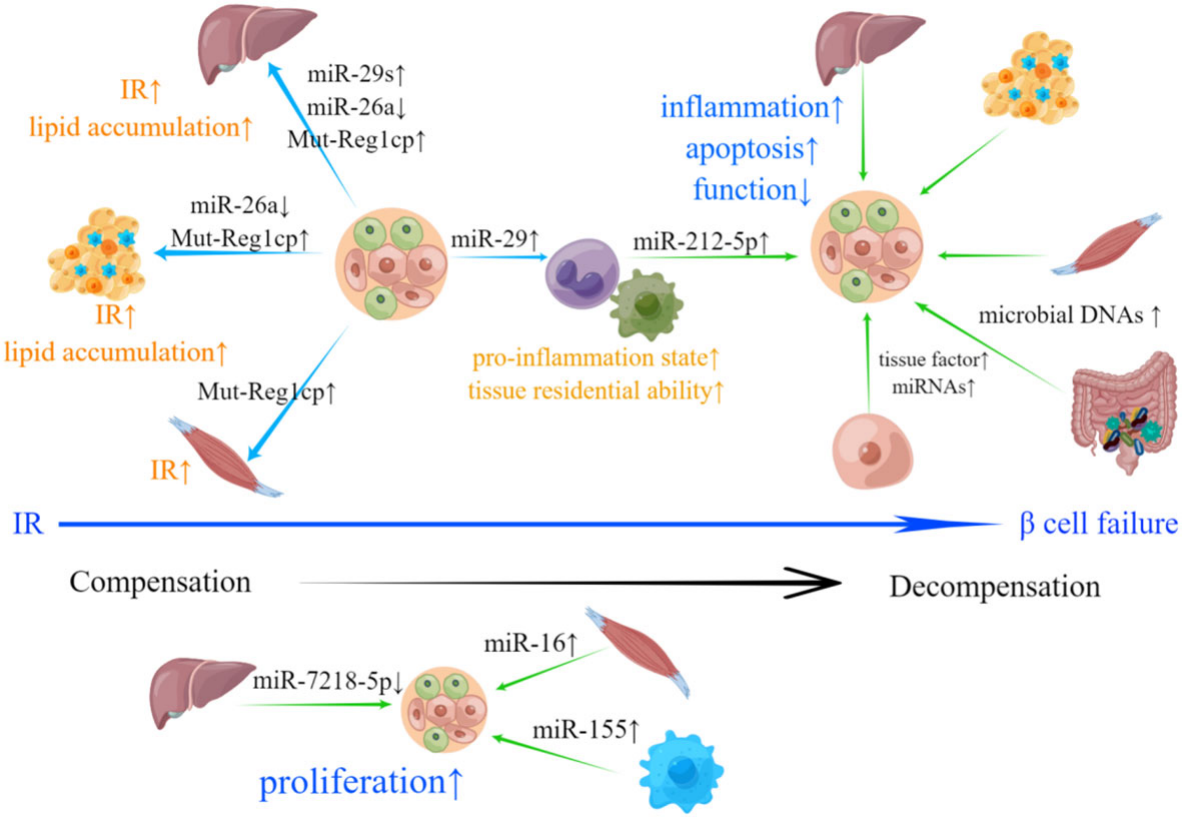
Involvement of islets- and peripheral tissue-derived EVs in T2DM-related pathological changes. IR is the hallmark of T2DM. EVs derived from β-cells can deliver bioactive materials including increased miR-29s, Mut-Reg1cp and reduced miR-26a to liver, adipose tissue and skeletal muscle, which results in IR and abnormal lipid accumulation. β-cells can also secrete EVs containing miR-29, promoting the transformation of monocytes and macrophages to an inflammatory phenotype and improving their tissue residential ability, which can lead to the exacerbation of IR. Upon the emergence of IR, pancreatic islets will compensate through hyperplasia. down-regulated miR-7218-5p, as well as up-regulated miR-16 and miR-155 loaded in EVs derived from liver, skeletal muscle and adipose tissue macrophages can promote β-cell proliferation. β-cell failure is a sign of systemic decompensation. EVs with bioactive materials shed by hepatocytes, adipose tissue, skeletal muscle, intestinal microorganism and M1 macrophage can induce inflammation, apoptosis, dysfunction and inhibit β-cell proliferation. EVs derived from β-cells with increased active procoagulant tissue factor and miRNAs may participate in the negative self-regulation of pancreatic islets.

In summary, EVs secreted by pancreatic islets (especially β-cells) and peripheral tissues play crucial roles in transmitting signals in the context of T2DM (**Figure 2**). β-cell-derived EVs can induce IR in peripheral tissues, while EVs derived from peripheral tissues can cause β-cell failure. Notably, β-cell-derived EVs can also act upon recipient β-cells and impose negative impacts in an autocrine manner in T2DM.

### 4.4 DM as secondary disease

The persistent infection of enteroviruses, especially Coxsackie virus, is considered to be associated with the appearance of T1DM, while its underlying mechanisms have not been fully elucidated (5). Geravandi et al. reported that under the condition of Coxsackie-viral infection, the transfer of β-cell-derived exosomal miRNAs to recipient cells may be associated with the inflammation, apoptosis and autoimmune response in pancreatic islets (111).

Cystic fibrosis is a type of genetic disease which is frequently accompanied by pulmonary infection and cystic fibrosis-related diabetes (CFRD) (112). CFRD is a unique type of DM that shares some features with both T1DM and T2DM (113). In the context of recurrent infection during cystic fibrosis, EVs derived from pancreatic exocrine cells can induce β-cell dysfunction and death *via* activating NF-κB pathway, which may be one of the pathogeneses of CFRD (6).

There is a prevailed incidence of type 3c DM in patients with pancreatic cancer, characterized by IR and hyperinsulinemia (114). EVs originated from pancreatic cancer cells can deliver adrenomedullin, CA19-9 and miR-19a to β-cells, which can induce ER stress and dysfunction of β-cells (7, 115). Moreover, pancreatic stellate cells (PSCs) play an important role in tumor growth and metastasis through creating the oncological microenvironment (116). The study by Pang et al. reported that EVs derived from PSCs can inhibit not only insulin secretion, but also proliferation of α-cells and β-cells, which may lead to type 3c DM as secondary disease of pancreatic cancer (117).

In conclusion, EVs emerge as novel vehicles transmitting signals *via* multifaceted cargos including miRNAs, inflammatory factors, adipokines, extracellular matrix proteins in the context of DM.

## 5 EVs serve as biomarkers and therapeutic agents for diagnosis and treatment of DM

As previously described, EVs serve as carriers transmitting signals to facilitate crosstalk of pancreatic islets in the context of physiology and DM. In addition, EVs are also viewed as potential biomarkers and promising therapeutic agents for diagnosis and treatment of DM (118, 119). EVs can be used as biomarkers for diagnosis due to their stability in easily accessible biological fluids, rapid and effective isolation and detection, as well as feasibility of relatively long-term preservation (8). Moreover, given that EVs can exert effects on target cells and prevent degradation of inherent biologically active molecules, they can also function as therapeutic agents (120). Here, we mainly discuss the isolation, characterization and clinical potentials of EVs that can reflect the status of pancreatic islets as biomarkers or improve islet function and viability as therapeutic agents (**Table 2**).

**Table 2:** Administration of EVs as therapeutic agents for the treatment of DM.

Author	Type of study	Administration
**Bai et al. (** 54)	Ex-vivo *In-vivo*	(1) Human iPSCs (5000 cells per well) were incubated with purified EVs (15 µg/mL) from 1 × 10^5^ cells for 15 days. (2) i-Beta cells (2 × 10^6^ cells/mouse) were transplanted under the kidney capsule of STZ-induced diabetic mice.
**Mahdipour et al. (** 55)	*In-vivo*	NR
**Mostafa-Hedeab et al. (** 56)	*In-vivo*	MSCs derived exosomes (0.4 µg/mL, 100µL, twice-weekly) were transplanted *via* tail vein of diabetic mice.
**Caxaria et al. (** 57)	Ex-vivo	high concentration (2x10^6^ particles/ml) intermediate concentration (5x10^5^ particles/ml) lower concentration (4x10^5^ particles/ml)
**Kalivarathan et al. (** 58)	Ex-vivo	NR
**Zhu et al. (** 59)	Ex-vivo	INS-1 cells (plated in six-well plates) were incubated with exosomes (10 µg).
**Tang et al. (** 60)	Ex-vivo	NR
**Sun et al. (** 25)	*In-vivo*	Exosomes (6×10^9^ particles) were transplanted *in situ* pancreas after low dose STZ injection, and exosomes (3×10^9^ particles) were infused 11 days after first transplantation through tail vein injection.
**Garcia-Contreras et al. (** 61)	Ex-vivo	NR
**Keshtkar et al. (** 62)	Ex-vivo	Islets (400 IEQ of islets per well) were incubated with exosomes (40 µg/ml) for three days.
**Mohammadi et al. (** 63)	*In-vivo*	Hybrid alginate microcapsule (AlgXO) loaded with exosomes (total number of exosomes within ~1000 AlgXO was 5.43 × 10^9^ ± 4.84 × 10^9^) and islets (1500 IEQ in total, islet equivalent) were transplanted into the intraperitoneal cavity of STZ-treated mice.
**Nie et al. (** 64)	Ex-vivo	NR

### 5.1 Insolation and characterization of EVs

Despite various differences between exosomes and MVs, most of current methods cannot completely separate them because they overlap in size, which results in low purity (121). So far, several isolation technologies have been established for efficient enrichment of exosomes or MVs, including commonly used ultracentrifugation (UC), polymer precipitation, size exclusion chromatography (SEC), ultrafiltration and immunoprecipitation (122–126) (1). UC mainly harvests the required components based on the size and density differences, typically separating the original solution into pellet cells and debris, large EVs and small EVs through a sequential increase in centrifugal force (2). Polymer precipitation usually uses polyethylene glycol (PEG) as a medium to reduce the solubility of EVs for precipitation and harvest. (3) SEC respectively separates macromolecules outside the gel pores and small molecules inside the gel pores by using a column. (4) Ultrafiltration selectively isolates samples through ultrafiltration membranes with different molecular weight cutoffs (MWCO). (5) Immunoprecipitation captures desired substances from heterogeneous mixtures based on the specific binding of monoclonal antibodies and ligands on the surface of EVs (122–126).

After isolation, EVs need to be characterized for their intended downstream application. In general, EV characterization methods are mainly categorized into two types: external characterization and inclusion characterization (127). We can detect morphology and particle size for external characterization through scanning electron microscopy (SEM), transmission electron microscopy (TEM), dynamic light-scattering (DLS) and nanoparticle tracking analysis (NTA) (128). As for inclusion characterization, western blotting, enzyme-linked immunosorbent assays and flow cytometry are optional methods to characterize membrane protein, lipid raft and other components of EVs, using classical biomarkers such as CD9, CD63, CD81, ALIX, and TSG101 (129–131). Notably, some new characterization methods have been reported recently. For instance, Islam et al. provided a way to characterize EVs by using nanoparticle-based time resolved fluorescence immunoassay, which can simplify the characterization step without extensive pretreatment (132). Paganini et al. presented a microfluidic device combining diffusion sizing and multiwavelength fluorescence detection to simultaneously provide information on EV size, concentration, and composition (133).

### 5.2 EVs as biomarkers reflecting the status of pancreatic islets

In the context of T1DM, profiling of RNAs from human islet-derived exosomes proven that coding and noncoding RNAs were differentially expressed under proinflammatory cytokines stress conditions and these RNAs were associated with insulin secretion, necrosis, apoptosis, and calcium signaling (134). The study provided a comprehensive catalog of RNAs from pancreatic islets that may serve as potential biomarkers for T1DM diagnosis (134). Lakhter et al. reported that increased miR-21-5p cargo in β-cell derived EVs in response to inflammatory cytokines can serve as a potential biomarker of T1DM (24). In T2DM conditions, miR-26a was down-regulated in islets of obese mice and reduced in serum exosomes in both obese mice and overweight humans, which was inversely correlated with clinical features of T2DM (94). Moreover, miR-375-3p was up-regulated in islet-derived exosomes after mixed cytokines or STZ treatment and dramatically increased in serum exosomes of STZ injected mice and new-onset T1DM and T2DM patients, which may serve as a potential biomarker of islets damage (135).

### 5.3 EVs as therapeutic agents improving function and viability of pancreatic islets

Autoimmune response-mediated destruction of the structure and function of pancreatic islets is identified as the main pathogenesis of T1DM (136). Strategies to replace, regenerate or promote the function of β-cells while inhibiting autoimmune response may allow for effective treatment (137). A study by Bai et al. showed that miR-212/132-enriched EVs derived from mature β-cells can promote differentiation of induced pluripotent stem cells into functional pancreatic β-cells (54), suggesting a novel approach of β-cell regeneration and replacement in the treatment of T1DM. MSCs are considered as ideal candidates to treat T1DM for their ability to promote β-cell function and regeneration (138, 139). It has been proven that EVs originated from MSCs can be delivered to β-cells to promote regeneration, insulin secretion and inhibit apoptosis, while the effects of MSCs-derived EVs on β-cells present a concentration-dependent manner (55–57). Advanced T1DM is also accompanied by dysfunction of α-cells, which may lead to severe hypoglycemia (140). Kalivarathan et al. found that under low-glucose condition, β-cell-derived EVs can significantly elevate the release of glucagon from α-cells, which may become one of the strategies for relieving severe hypoglycemia in advanced T1DM (58).

Pancreatic β-cell failure is one of main hallmarks in T2DM (141). Thus, maintaining the mass and function of β-cells to halt the progressive β-cell failure is an effective therapy to treat T2DM (142). A study by Zhu et al. showed that EVs derived from low-dose cytokine-induced β-cells can prevent high-dose cytokine-triggered β-cell death through the action of cargo neutral ceramidase (NCDase) (59, 60). MSCs-derived EVs can promote β-cell function and regeneration while inhibit apoptosis, serving as potential treatment strategy for T2DM (25). In a low-dose STZ-induced diabetic animal model, EVs derived from β-cells under high-glucose stimulation can reduce macrophage infiltration and enhance the expression of CD31, a marker of endothelial cells in islets, playing key roles in preserving pancreatic islet architecture and function (143).

Islet transplantation has become an established treatment option for insulin-deficient DM (144). The pre-transplantation status of islets and post-transplantation injury factors including hypoxia and immune rejection can dramatically influence the effects of clinical operation (145). Therefore, strategies to improve islets mass and function prior to transplantation and prevent hypoxia and immune response-mediated graft rejection after transplantation may be the key to improve therapeutic effects (146, 147). Pancreatic islets treated with EVs derived from umbilical vein endothelial cells present better structure and function compared with untreated group, which provides a new method of improving pre-transplantation status of islets (61). EVs containing vascular endothelial growth factor (VEGF) derived from MSCs are also shown to promote the survival and function of the isolated islets (62). Furthermore, EVs derived from MSCs can reduce immune rejection, inflammation, fibrosis of pancreatic islets and increase hypoxic resistance *via* ROS-NLRP3-TXNIP pathway after transplantation (63, 64).

## 6 Conclusion

Pancreatic islets play vital roles in maintenance of glucose homeostasis by secreting various hormones, whose dysfunction causes DM (148). EVs not only act as intercellular communication vehicle, but also serve as potential biomarkers and therapeutic agents for the diagnosis and treatment of human diseases (149). In this review, we provided an overview of EVs with the focus on EV generation and cellular recognition and uptake of EVs, discussed EVs-mediated intra-islet crosstalk as well as crosstalk between pancreatic islets and extra-islet tissues under physiological and diabetic conditions, summarized the emerging applications of EVs in the treatment of DM.

Although much effects have been made, the underlying mechanisms of EVs-mediated intercellular and interorgan crosstalk of pancreatic islets are still largely unknown. For instance, it is still unclear how the expression of EVs cargo is regulated, how EVs cargo is selectively packaged, and how EVs can be recognized and taken up by specific cell types. Pancreatic islets are composed of a variety of endocrine cells including insulin-secreting β-cells, glucagon-secreting α-cells and somatostatin-secreting δ-cells, which tightly regulate blood glucose homeostasis (17). Accumulating evidence suggest that non-β islet cells, such as α-cells and δ-cells, are also involved in the dysregulated cellular communication and the development of DM (150–152). Hence, more studies are needed to understand EVs-mediated intercellular and interorgan crosstalk of non-beta islet cells under both physiological and pathological conditions. In addition, a wide range of cargos packaged in EVs play a role in the pathophysiology of DM, including regulatory, inflammatory, metabolic, and cellular cargos. While most current research on EVs-mediated crosstalk between islets and extra-islet tissues focused on the role of miRNAs, it is important for future studies to pay attention to cargos beyond miRNAs. Given the critical roles of brain-islet axis in glucose homeostasis and DM, as well as the strong association between DM and neurodegenerative diseases, it is also meaningful to investigate the roles of EVs in mediating brain-islet crosstalk in this context (153–155).

Collective evidence suggests that EVs derived from various cell sources may serve as therapeutic agents in the treatment of DM (149). In the current preclinical studies on EVs, there is a lack of detail investigation on appropriate dosage, frequency, and timing of EV administration. Moreover, a uniform definition for reversal of DM has not been established, highlighting the need for future improvement in this field. Meanwhile, the clinical application of EVs is hindered by its non-specific organ accumulation and relative instability (156). Yerneni et al. reported that EVs can be engineered to acquire capability of targeting through oligonucleotide tethers without changing inherent properties of EVs (157). Rayamajhi et al. reported that EVs can be hybridized with synthetic liposome to generate hybrid exosomes for preventing poor yield and dysfunction during isolation (158). EVs-based therapeutics for DM may be significantly improved through the integration of multidisciplinary technologies. Besides, the current research on the plasma half-life of EVs and whether they can induce intensive antibody responses is controversial and requires further investigation (159, 160).

## Author contributions

Conceptualization, XiaZ. Writing—original draft preparation, JW, ZW, TH, and JC. Writing—review and editing, YO, LW, XiaZ, KW, ZY, Y-PH, and XiaZ. All authors have read and agreed to the published version of the manuscript.

## References

[1] Khan RMM Chua ZJY JC T Yang Y Liao Z Zhao Y. From pre-diabetes to diabetes: diagnosis, treatments and translational research. Medicina (Kaunas) (2019) 55(9):546. doi: 10.3390/medicina55090546 31470636 PMC6780236

[2] Sun H Saeedi P Karuranga S Pinkepank M Ogurtsova K BB D. Idf diabetes atlas: global, regional and country-level diabetes prevalence estimates for 2021 and projections for 2045. Diabetes Res Clin Pract (2022) 183:109119. doi: 10.1016/j.diabres.2021.109119 34879977 PMC11057359

[3] Mauricio D Alonso N Gratacos M. Chronic diabetes complications: the need to move beyond classical concepts. Trends Endocrinol Metab (2020) 31(4):287–95. doi: 10.1016/j.tem.2020.01.007 32033865

[4] Brody H. Diabetes. Nature (2012) 485(7398):S1. doi: 10.1038/485s1a 22616093

[5] Nekoua MP Alidjinou EK Hober D. Persistent coxsackievirus b infection and pathogenesis of type 1 diabetes mellitus. Nat Rev Endocrinol (2022) 18(8):503–16. doi: 10.1038/s41574-022-00688-1 PMC9157043 35650334

[6] Constantinescu AA Gleizes C Alhosin M Yala E Zobairi F Leclercq A. Exocrine cell-derived microparticles in response to lipopolysaccharide promote endocrine dysfunction in cystic fibrosis. J Cyst Fibros (2014) 13(2):219–26. doi: 10.1016/j.jcf.2013.08.012 24095207

[7] Javeed N Sagar G Dutta SK Smyrk TC Lau JS Bhattacharya S. Pancreatic cancer-derived exosomes cause paraneoplastic β-cell dysfunction. Clin Cancer Res (2015) 21(7):1722–33. doi: 10.1158/1078-0432.Ccr-14-2022 PMC4383684 25355928

[8] Pardo F Villalobos-Labra R Sobrevia B Toledo F Sobrevia L. Extracellular vesicles in obesity and diabetes mellitus. Mol Aspects Med (2018) 60:81–91. doi: 10.1016/j.mam.2017.11.010 29175307

[9] Colombo M Raposo G Thery C. Biogenesis, secretion, and intercellular interactions of exosomes and other extracellular vesicles. Annu Rev Cell Dev Biol (2014) 30:255–89. doi: 10.1146/annurev-cellbio-101512-122326 25288114

[10] Kalluri R LeBleu VS. The biology, function, and biomedical applications of exosomes. Science (2020) 367(6478):eaau6977. doi: 10.1126/science.aau6977 32029601 PMC7717626

[11] van Niel G D'Angelo G Raposo G. Shedding light on the cell biology of extracellular vesicles. Nat Rev Mol Cell Biol (2018) 19(4):213–28. doi: 10.1038/nrm.2017.125 29339798

[12] Li J Zhang Y Jiang X. Pancreatic islet-secreted exosomal microrna-29 family members travel to liver and promote hepatic insulin resistance. J Extracell Vesicles (2018) 7(Supplement 1):252–3. doi: 10.1080/20013078.2018.1461450

[13] de Abreu RC Fernandes H da Costa Martins PA Sahoo S Emanueli C Ferreira L. Native and bioengineered extracellular vesicles for cardiovascular therapeutics. Nat Rev Cardiol (2020) 17(11):685–97. doi: 10.1038/s41569-020-0389-5 PMC7874903 32483304

[14] Sadovska L Eglitis J Line A. Extracellular vesicles as biomarkers and therapeutic targets in breast cancer. Anticancer Res (2015) 35(12):6379–90. 26637847

[15] Liu H Li R Liu T Yang L Yin G Xie Q. Immunomodulatory effects of mesenchymal stem cells and mesenchymal stem cell-derived extracellular vesicles in rheumatoid arthritis. Front Immunol (2020) 11:1912. doi: 10.3389/fimmu.2020.01912 32973792 PMC7468450

[16] Gratpain V Mwema A Labrak Y Muccioli GG van Pesch V des Rieux A. Extracellular vesicles for the treatment of central nervous system diseases. Adv Drug Delivery Rev (2021) 174:535–52. doi: 10.1016/j.addr.2021.05.006 33991589

[17] Campbell JE Newgard CB. Mechanisms controlling pancreatic islet cell function in insulin secretion. Nat Rev Mol Cell Biol (2021) 22(2):142–58. doi: 10.1038/s41580-020-00317-7 PMC8115730 33398164

[18] Walker JT Saunders DC Brissova M Powers AC. The human islet: mini-organ with mega-impact. Endocr Rev (2021) 42(5):605–57. doi: 10.1210/endrev/bnab010 PMC8476939 33844836

[19] Harcourt BE Penfold SA Forbes JM. Coming full circle in diabetes mellitus: from complications to initiation. Nat Rev Endocrinol (2013) 9(2):113–23. doi: 10.1038/nrendo.2012.236 23296171

[20] Petersen MC Shulman GI. Mechanisms of insulin action and insulin resistance. Physiol Rev (2018) 98(4):2133–223. doi: 10.1152/physrev.00063.2017 PMC6170977 30067154

[21] Gesmundo I Pardini B Gargantini E Gamba G Birolo G Fanciulli A. Adipocyte-derived extracellular vesicles regulate survival and function of pancreatic β cells. JCI Insight (2021) 6(5). doi: 10.1172/jci.insight.141962 PMC8021102 33539327

[22] Ribeiro D Horvath I Heath N Hicks R Forslöw A Wittung-Stafshede P. Extracellular vesicles from human pancreatic islets suppress human islet amyloid polypeptide amyloid formation. Proc Natl Acad Sci U.S.A. (2017) 114(42):11127–32. doi: 10.1073/pnas.1711389114 PMC5651775 28973954

[23] Castano C Novials A Parrizas M. Exosomes and diabetes. Diabetes Metab Res Rev (2019) 35(3):e3107. doi: 10.1002/dmrr.3107 30513130

[24] Lakhter AJ Pratt RE Moore RE Doucette KK Maier BF DiMeglio LA. Beta cell extracellular vesicle mir-21-5p cargo is increased in response to inflammatory cytokines and serves as a biomarker of type 1 diabetes. Diabetologia (2018) 61(5):1124–34. doi: 10.1007/s00125-018-4559-5 PMC5878132 29445851

[25] Sun Y Shi H Yin S Ji C Zhang X Zhang B. Human mesenchymal stem cell derived exosomes alleviate type 2 diabetes mellitus by reversing peripheral insulin resistance and relieving β-cell destruction. ACS Nano (2018) 12(8):7613–28. doi: 10.1021/acsnano.7b07643 30052036

[26] Meldolesi J. Exosomes and ectosomes in intercellular communication. Curr Biol (2018) 28(8):R435–R44. doi: 10.1016/j.cub.2018.01.059 29689228

[27] Thery C Witwer KW Aikawa E Alcaraz MJ Anderson JD Andriantsitohaina R. Minimal information for studies of extracellular vesicles 2018 (Misev2018): a position statement of the international society for extracellular vesicles and update of the Misev2014 guidelines. J Extracell Vesicles (2018) 7(1):1535750. doi: 10.1080/20013078.2018.1535750 30637094 PMC6322352

[28] Ma L Li Y Peng J Wu D Zhao X Cui Y. Discovery of the migrasome, an organelle mediating release of cytoplasmic contents during cell migration. Cell Res (2015) 25(1):24–38. doi: 10.1038/cr.2014.135 25342562 PMC4650581

[29] Hessvik NP Øverbye A Brech A ML T IS J Sandvig K. Pikfyve inhibition increases exosome release and induces secretory autophagy. Cell Mol Life Sci CMLS (2016) 73(24):4717–37. doi: 10.1007/s00018-016-2309-8 PMC11108566 27438886

[30] Melentijevic I Toth ML Arnold ML Guasp RJ Harinath G Nguyen KC. C. elegans neurons jettison protein aggregates and mitochondria under neurotoxic stress. Nature (2017) 542(7641):367–71. doi: 10.1038/nature21362 PMC5336134 28178240

[31] Santavanond JP Rutter SF Atkin-Smith GK Poon IKH. Apoptotic bodies: mechanism of formation, isolation and functional relevance. Sub-cellular Biochem (2021) 97:61–88. doi: 10.1007/978-3-030-67171-6_4 33779914

[32] Ashley J Cordy B Lucia D Fradkin LG Budnik V Thomson T. Retrovirus-like gag protein Arc1 binds rna and traffics across synaptic boutons. Cell (2018) 172(1-2):262–74. doi: 10.1016/j.cell.2017.12.022 PMC5793882 29328915

[33] Al-Nedawi K Meehan B Micallef J Lhotak V May L Guha A. Intercellular transfer of the oncogenic receptor egfrviii by microvesicles derived from tumour cells. Nat Cell Biol (2008) 10(5):619–24. doi: 10.1038/ncb1725 18425114

[34] Hurley JH. Escrt complexes and the biogenesis of multivesicular bodies. Curr Opin Cell Biol (2008) 20(1):4–11. doi: 10.1016/j.ceb.2007.12.002 18222686 PMC2282067

[35] Stuffers S Sem Wegner C Stenmark H Brech A. Multivesicular endosome biogenesis in the absence of escrts. Traffic (2009) 10(7):925–37. doi: 10.1111/j.1600-0854.2009.00920.x 19490536

[36] Bonifacino JS Glick BS. The mechanisms of vesicle budding and fusion. Cell (2004) 116(2):153–66. doi: 10.1016/s0092-8674(03)01079-1 14744428

[37] Cai H Reinisch K Ferro-Novick S. Coats, tethers, rabs, and snares work together to mediate the intracellular destination of a transport vesicle. Dev Cell (2007) 12(5):671–82. doi: 10.1016/j.devcel.2007.04.005 17488620

[38] Jahn R Scheller RH. Snares–engines for membrane fusion. Nat Rev Mol Cell Biol (2006) 7(9):631–43. doi: 10.1038/nrm2002 16912714

[39] Rilla K. Diverse plasma membrane protrusions act as platforms for extracellular vesicle shedding. J Extracell vesicles (2021) 10(11):e12148. doi: 10.1002/jev2.12148 34533887 PMC8448080

[40] Minciacchi VR Freeman MR Di Vizio D. Extracellular vesicles in cancer: exosomes, microvesicles and the emerging role of Large oncosomes. Semin Cell Dev Biol (2015) 40:41–51. doi: 10.1016/j.semcdb.2015.02.010 25721812 PMC4747631

[41] Li B Antonyak MA Zhang J Cerione RA. Rhoa triggers a specific signaling pathway that generates transforming microvesicles in cancer cells. Oncogene (2012) 31(45):4740–9. doi: 10.1038/onc.2011.636 PMC3607381 22266864

[42] Warburg O. On respiratory impairment in cancer cells. Science (1956) 124(3215):269–70. doi: 10.1126/science.124.3215.269 13351639

[43] Mathieu M Martin-Jaular L Lavieu G Théry C. Specificities of secretion and uptake of exosomes and other extracellular vesicles for cell-to-Cell communication. Nat Cell Biol (2019) 21(1):9–17. doi: 10.1038/s41556-018-0250-9 30602770

[44] Cheng L Sun X Scicluna BJ Coleman BM Hill AF. Characterization and deep sequencing analysis of exosomal and non-exosomal mirna in human urine. Kidney Int (2014) 86(2):433–44. doi: 10.1038/ki.2013.502 24352158

[45] Trajkovic K Hsu C Chiantia S Rajendran L Wenzel D Wieland F. Ceramide triggers budding of exosome vesicles into multivesicular endosomes. Science (2008) 319(5867):1244–7. doi: 10.1126/science.1153124 18309083

[46] Villarroya-Beltri C Baixauli F Gutiérrez-Vázquez C Sánchez-Madrid F Mittelbrunn M. Sorting it out: regulation of exosome loading. Semin Cancer Biol (2014) 28:3–13. doi: 10.1016/j.semcancer.2014.04.009 24769058 PMC4640178

[47] Raposo G Stoorvogel W. Extracellular vesicles: exosomes, microvesicles, and friends. J Cell Biol (2013) 200(4):373–83. doi: 10.1083/jcb.201211138 PMC3575529 23420871

[48] Maas SLN Breakefield XO Weaver AM. Extracellular vesicles: unique intercellular delivery vehicles. Trends Cell Biol (2017) 27(3):172–88. doi: 10.1016/j.tcb.2016.11.003 PMC5318253 27979573

[49] Christianson HC Svensson KJ Belting M. Exosome and microvesicle mediated phene transfer in mammalian cells. Semin Cancer Biol (2014) 28:31–8. doi: 10.1016/j.semcancer.2014.04.007 24769057

[50] Mulcahy LA Pink RC Carter DR. Routes and mechanisms of extracellular vesicle uptake. J Extracell Vesicles (2014) 3:24641. doi: 10.3402/jev.v3.24641 PMC4122821 25143819

[51] Tian T Zhu YL Hu FH Wang YY Huang NP Xiao ZD. Dynamics of exosome internalization and trafficking. J Cell Physiol (2013) 228(7):1487–95. doi: 10.1002/jcp.24304 23254476

[52] Chen W Wen X Latzel M Heilmann M Yang J Dai X. Nanoscale characterization of carrier dynamic and surface passivation in Ingan/Gan multiple quantum wells on gan nanorods. ACS Appl Mater Interfaces (2016) 8(46):31887–93. doi: 10.1021/acsami.6b11675 27797477

[53] Tian T Wang Y Wang H Zhu Z Xiao Z. Visualizing of the cellular uptake and intracellular trafficking of exosomes by live-cell microscopy. J Cell Biochem (2010) 111(2):488–96. doi: 10.1002/jcb.22733 20533300

[54] Bai C Ren Q Liu H Li X Guan W Gao Y. Mir-212/132-Enriched extracellular vesicles promote differentiation of induced pluripotent stem cells into pancreatic beta cells. Front Cell Dev Biol (2021) 9:673231. doi: 10.3389/fcell.2021.673231 34055806 PMC8155495

[55] Mahdipour E Salmasi Z Sabeti N. Exosomes isolated from menstrual blood-derived mesenchymal stem cells regenerate the beta cell mass in diabetic type 1 animal model. BioImpacts (2018) 8(Supplement 1):41–2. doi: 10.15171/bi.2018.S1

[56] Mostafa-Hedeab G Hassan ME Sabry D Ali RI. Anti-diabetic therapeutic efficacy of mesenchymal stem cells-derived exosomes. Int J Pharmacol (2020) 16(6):437–46. doi: 10.3923/ijp.2020.437.446

[57] Caxaria S Rackham C Aziz T King A Jones P. Mesenchymal stromal cell derived exosomes improve islet function and survival. Diabetologia (2020) 63(SUPPL 1):S189–S90. doi: 10.1007/s00125-020-05221-5

[58] Kalivarathan J Saravanan PB Levy MF Kanak MA. Regulation of alpha-cell function by beta-cell extracellular vesicles. Diabetes Conference: 80th Sci Sessions Am Diabetes Association ADA (2020) 69(Supplement 1). doi: 10.2337/db20-2102-P

[59] Zhu Q Kang J Miao H Feng Y Xiao L Hu Z. Low-dose cytokine-induced neutral ceramidase secretion from ins-1 cells *Via* exosomes and its anti-apoptotic effect. FEBS J (2014) 281(12):2861–70. doi: 10.1111/febs.12826 24798654

[60] Tang S Luo F Feng YM Wei X Miao H Lu YB. Neutral ceramidase secreted via exosome protects against palmitate-induced apoptosis in INS-1 cells. Exp Clin Endocrinol Diabetes (2017) 125(02):130–5. doi: 10.1055/s-0042-116314 28008587

[61] Garcia-Contreras M Mendez AJ Ricordi C. Enhancement of pre-transplant in-vitro culture of human pancreatic islets by supplementation with human endothelial-derived exosomes. Xenotransplantation (2015) 1):S170–S1. doi: 10.1111/xen.12206

[62] Keshtkar S Kaviani M Sarvestani FS Ghahremani MH Aghdaei MH Al-Abdullah IH. Exosomes derived from human mesenchymal stem cells preserve mouse islet survival and insulin secretion function. EXCLI J (2020) 19:1064–80. doi: 10.17179/excli2020-2451 PMC7527509 33013264

[63] Mohammadi MR Rodriguez SM Luong JC Li S Cao R Alshetaiwi H. Exosome loaded immunomodulatory biomaterials alleviate local immune response in immunocompetent diabetic mice post islet xenotransplantation. Commun Biol (2021) 4(1):685. doi: 10.1038/s42003-021-02229-4 34083739 PMC8175379

[64] Nie W Chen C Ma X Wang W. (2019). Human mesenchymal stem cell-derived exosomes enhances porcine islets resistance to hypoxia via suppression of Nlrp3 inflammasome activation. Xenotransplantation (NJ USA: Wiley) 26:5.

[65] Zhang A Li D Liu Y Li J Zhang Y Zhang CY. Islet β cell: an endocrine cell secreting mirnas. Biochem Biophys Res Commun (2018) 495(2):1648–54. doi: 10.1016/j.bbrc.2017.12.028 29223394

[66] Cypess AM. Reassessing human adipose tissue. New Engl J Med (2022) 386(8):768–79. doi: 10.1056/NEJMra2032804 35196429

[67] Hernandez MG Aguilar AG Burillo J Oca RG Manca MA Novials A. Pancreatic beta cells overexpressing hiapp impaired mitophagy and unbalanced mitochondrial dynamics. Cell Death Dis (2018) 9(5):481. doi: 10.1038/s41419-018-0533-x 29705815 PMC5924657

[68] Raleigh D Zhang X Hastoy B Clark A. The β-cell assassin: iapp cytotoxicity. J Mol Endocrinol (2017) 59(3):R121–r40. doi: 10.1530/jme-17-0105 28811318

[69] Shen Z Jiang H HT H Qian L Fu Q Shen M. Microrna-127 inhibits cell proliferation *Via* targeting Kif3b in pancreatic β cells. Aging (Albany NY) (2019) 11(5):1342–55. doi: 10.18632/aging.101835 PMC6428088 30822278

[70] Syed FZ. Type 1 diabetes mellitus. Ann Intern Med (2022) 175(3):ITC33–48. doi: 10.7326/AITC202203150 35254878

[71] Katsarou A Gudbjornsdottir S Rawshani A Dabelea D Bonifacio E Anderson BJ. Type 1 diabetes mellitus. Nat Rev Dis Primers (2017) 3:1–17. doi: 10.1038/nrdp.2017.16 28358037

[72] Grieco GE Fignani D Formichi C Nigi L Licata G Maccora C. Extracellular vesicles in immune system regulation and type 1 diabetes: cell-to-Cell communication mediators, disease biomarkers, and promising therapeutic tools. Front Immunol (2021) 12:682948. doi: 10.3389/fimmu.2021.682948 34177928 PMC8219977

[73] Hasilo CP Negi S Allaeys I Cloutier N Rutman AK Gasparrini M. Presence of diabetes autoantigens in extracellular vesicles derived from human islets. Sci Rep (2017) 7(1):5000. doi: 10.1038/s41598-017-04977-y 28694505 PMC5504025

[74] Cianciaruso C Phelps EA Pasquier M Hamelin R Demurtas D Alibashe Ahmed M. Primary human and rat β-cells release the intracellular autoantigens Gad65, ia-2, and proinsulin in exosomes together with cytokine-induced enhancers of immunity. Diabetes (2017) 66(2):460–73. doi: 10.2337/db16-0671 27872147

[75] Sheng H Hassanali S Nugent C Wen L Hamilton-Williams E Dias P. Insulinoma-released exosomes or microparticles are immunostimulatory and can activate autoreactive T cells spontaneously developed in nonobese diabetic mice. J Immunol (2011) 187(4):1591–600. doi: 10.4049/jimmunol.1100231 PMC3150365 21734072

[76] Rahman MJ Regn D Bashratyan R Dai YD. Exosomes released by islet-derived mesenchymal stem cells trigger autoimmune responses in nod mice. Diabetes (2014) 63(3):1008–20. doi: 10.2337/db13-0859 PMC3931393 24170696

[77] Vomund AN Zinselmeyer BH Hughes J Calderon B Valderrama C Ferris ST. Beta cells transfer vesicles containing insulin to phagocytes for presentation to T cells. Proc Natl Acad Sci U.S.A. (2015) 112(40):E5496–502. doi: 10.1073/pnas.1515954112 PMC4603448 26324934

[78] Rutman AK Negi S Gasparrini M CP H Tchervenkov J Paraskevas S. Immune response to extracellular vesicles from human islets of langerhans in patients with type 1 diabetes. Endocrinology (2018) 159(11):3834–47. doi: 10.1210/en.2018-00649 30307543

[79] Tesovnik T Kovac J Pohar K Hudoklin S Dovc K Bratina N. Extracellular vesicles derived human-mirnas modulate the immune system in type 1 diabetes. Front Cell Dev Biol (2020) 8:202. doi: 10.3389/fcell.2020.00202 32296701 PMC7136501

[80] Giri KR de Beaurepaire L Jegou D Lavy M Mosser M Dupont A. Molecular and functional diversity of distinct subpopulations of the stressed insulin-secreting cell's vesiculome. Front Immunol (2020) 11:1814. doi: 10.3389/fimmu.2020.01814 33101266 PMC7556286

[81] Brozzi F Cosentino C Jacovetti C Wu K Menoud V Bayazit MB. Role of trna-derived fragments in the cross-talk between immune cells and beta cells during type 1 diabetes pathogenesis. Diabetologia (2022) 65(Supplement 1):S57. doi: 10.1007/s00125-022-05755-w PMC11446995 38967669

[82] Guay C Kruit JK Rome S Menoud V Mulder NL Jurdzinski A. Lymphocyte-derived exosomal micrornas promote pancreatic β cell death and may contribute to type 1 diabetes development. Cell Metab (2019) 29(2):348–61.e6. doi: 10.1016/j.cmet.2018.09.011 30318337

[83] Stumvoll M Goldstein BJ van Haeften TW. Type 2 diabetes: principles of pathogenesis and therapy. Lancet (2005) 365(9467):1333–46. doi: 10.1016/S0140-6736(05)61032-X 15823385

[84] Roden M Shulman GI. The integrative biology of type 2 diabetes. Nature (2019) 576(7785):51–60. doi: 10.1038/s41586-019-1797-8 31802013

[85] Donath MY. Targeting inflammation in the treatment of type 2 diabetes: time to start. Nat Rev Drug Discovery (2014) 13(6):465–76. doi: 10.1038/nrd4275 24854413

[86] Ghorpade DS Ozcan L Zheng Z Nicoloro SM Shen Y Chen E. Hepatocyte-secreted Dpp4 in obesity promotes adipose inflammation and insulin resistance. Nature (2018) 555(7698):673–7. doi: 10.1038/nature26138 PMC6021131 29562231

[87] Savage DB Petersen KF Shulman GI. Disordered lipid metabolism and the pathogenesis of insulin resistance. Physiol Rev (2007) 87(2):507–20. doi: 10.1152/physrev.00024.2006 PMC2995548 17429039

[88] Mezza T Cinti F Cefalo CMA Pontecorvi A RN K Giaccari A. β-cell fate in human insulin resistance and type 2 diabetes: a perspective on islet plasticity. Diabetes (2019) 68(6):1121–9. doi: 10.2337/db18-0856 PMC6905483 31109941

[89] Liu J Zhang Y Tian Y Huang W Tong N Fu X. Integrative biology of extracellular vesicles in diabetes mellitus and diabetic complications. Theranostics (2022) 12(3):1342–72. doi: 10.7150/thno.65778 PMC8771544 35154494

[90] Ozcan U Cao Q Yilmaz E AH L NN I Ozdelen E. Endoplasmic reticulum stress links obesity, insulin action, and type 2 diabetes. Science (2004) 306(5695):457–61. doi: 10.1126/science.1103160 15486293

[91] Lee YS Olefsky J. Chronic tissue inflammation and metabolic disease. Genes Dev (2021) 35(5-6):307–28. doi: 10.1101/gad.346312.120 PMC7919414 33649162

[92] Guevara-Olaya L Chimal-Vega B Castaneda-Sanchez CY Lopez-Cossio LY Pulido-Capiz A Galindo-Hernandez O. Ldl promotes disorders in beta-cell cholesterol metabolism, implications on insulin cellular communication mediated by evs. Metabolites (2022) 12(8):754. doi: 10.3390/metabo12080754 36005626 PMC9415214

[93] Li J Zhang Y Ye Y Li D Liu Y Lee E. Pancreatic β cells control glucose homeostasis *Via* the secretion of exosomal mir-29 family. J Extracell Vesicles (2021) 10(3):e12055. doi: 10.1002/jev2.12055 33520119 PMC7820156

[94] Xu H Du X Xu J Zhang Y Tian Y Liu G. Pancreatic β cell microrna-26a alleviates type 2 diabetes by improving peripheral insulin sensitivity and preserving β cell function. PloS Biol (2020) 18(2):e3000603. doi: 10.1371/journal.pbio.3000603 32092075 PMC7058362

[95] Guo WH Guo Q Liu YL Yan DD Jin L Zhang R. Mutated lncrna increase the risk of type 2 diabetes by promoting beta cell dysfunction and insulin resistance. Cell Death Dis (2022) 13(10):904. doi: 10.1038/s41419-022-05348-w 36302749 PMC9613878

[96] Stenvers DJ Scheer F Schrauwen P la Fleur SE Kalsbeek A. Circadian clocks and insulin resistance. Nat Rev Endocrinol (2019) 15(2):75–89. doi: 10.1038/s41574-018-0122-1 30531917

[97] Sun Y Zhou Y Shi Y Zhang Y Liu K Liang R. Expression of mirna-29 in pancreatic β cells promotes inflammation and diabetes *Via* Traf3. Cell Rep (2021) 34(1):108576. doi: 10.1016/j.celrep.2020.108576 33406428

[98] Bouwens L Rooman I. Regulation of pancreatic beta-cell mass. Physiol Rev (2005) 85(4):1255–70. doi: 10.1152/physrev.00025.2004 16183912

[99] Fu Q Li Y Jiang H Shen Z Gao R He Y. Hepatocytes derived extracellular vesicles from high-fat diet induced obese mice modulate genes expression and proliferation of islet β cells. Biochem Biophys Res Commun (2019) 516(4):1159–66. doi: 10.1016/j.bbrc.2019.06.124 31284955

[100] Jalabert A Vial G Guay C Wiklander OP Nordin JZ Aswad H. Exosome-like vesicles released from lipid-induced insulin-resistant muscles modulate gene expression and proliferation of beta recipient cells in mice. Diabetologia (2016) 59(5):1049–58. doi: 10.1007/s00125-016-3882-y 26852333

[101] Gao H Luo Z Jin Z Ji Y Ying W. Adipose tissue macrophages modulate obesity-associated β cell adaptations through secreted mirna-containing extracellular vesicles. Cells (2021) 10(9):2451. doi: 10.3390/cells10092451 34572101 PMC8472266

[102] Alen R Cobo-Vuilleumier N Gauthier BR GarciaMartinez I Valverde AM. Effect of lipotoxic hepatocyte-derived extracellular vesicles in pancreas inflammation and beta cell functionality. Diabetologia (2022) 65(Supplement 1):S247–S8. doi: 10.1007/s00125-022-05755-w PMC12245964 40387904

[103] Rome S Forterre A Vial G Besse A Chikh K Coute Y. Muscle secreted exosomes: a new paradigm for musclepancreas cross talk? Obes Facts (2013) 1:18. doi: 10.1159/000250038

[104] Honda K Littman DR. The microbiota in adaptive immune homeostasis and disease. Nature (2016) 535(7610):75–84. doi: 10.1038/nature18848 27383982

[105] Ma Q Li Y Li P Wang M Wang J Tang Z. Research progress in the relationship between type 2 diabetes mellitus and intestinal flora. Biomedicine pharmacother = Biomedecine pharmacotherapie (2019) 117:109138. doi: 10.1016/j.biopha.2019.109138 31247468

[106] Gao H Luo Z Ji Y Tang K Jin Z Ly C. Accumulation of microbial dnas promotes to islet inflammation and β cell abnormalities in obesity in mice. Nat Commun (2022) 13(1):565. doi: 10.1038/s41467-022-28239-2 35091566 PMC8799656

[107] Qian B Yang Y Tang N Wang J Sun P Yang N. M1 macrophage-derived exosomes impair beta cell insulin secretion *Via* mir-212-5p by targeting Sirt2 and inhibiting Akt/Gsk-3β/β-Catenin pathway in mice. Diabetologia (2021) 64(9):2037–51. doi: 10.1007/s00125-021-05489-1 34117507

[108] Chidester S Livinski AA Fish AF Joseph PV. The role of extracellular vesicles in β-cell function and viability: a scoping review. Front Endocrinol (Lausanne) (2020) 11:375. doi: 10.3389/fendo.2020.00375 32595604 PMC7300279

[109] Gleizes C Kreutter G Abbas M Kassem M Constantinescu AA Boisramé-Helms J. β cell membrane remodelling and procoagulant events occur in inflammation-driven insulin impairment: a glp-1 receptor dependent and independent control. J Cell Mol Med (2016) 20(2):231–42. doi: 10.1111/jcmm.12683 PMC4727568 26607759

[110] Guay C Menoud V Rome S Regazzi R. Horizontal transfer of exosomal micrornas transduce apoptotic signals between pancreatic beta-cells. Cell Commun Signal (2015) 13:17. doi: 10.1186/s12964-015-0097-7 25880779 PMC4371845

[111] Geravandi S Dasgupta B Liu H Maedler K. Exosomal transfer of mirnas in response to coxsackie-viral infection in beta cells. Diabetologia (2020) 63(SUPPL 1):S158–S9. doi: 10.1007/s00125-020-05221-5

[112] Elborn JS. Cystic fibrosis. Lancet (London England) (2016) 388(10059):2519–31. doi: 10.1016/s0140-6736(16)00576-6 27140670

[113] Prentice BJ Potter KJ Coriati A Boudreau V Rusnell L Kherani T. Cystic fibrosis-related diabetes: clinical approach and knowledge gaps. Paediatric Respir Rev (2022). doi: 10.1016/j.prrv.2022.10.001 36376223

[114] Hart PA Bellin MD Andersen DK Bradley D Cruz-Monserrate Z Forsmark CE. Type 3c (Pancreatogenic) diabetes mellitus secondary to chronic pancreatitis and pancreatic cancer. Lancet Gastroenterol Hepatol (2016) 1(3):226–37. doi: 10.1016/s2468-1253(16)30106-6 PMC5495015 28404095

[115] Pang W Yao W Dai X Zhang A Hou L Wang L. Pancreatic cancer-derived exosomal microrna-19a induces β-cell dysfunction by targeting Adcy1 and Epac2. Int J Biol Sci (2021) 17(13):3622–33. doi: 10.7150/ijbs.56271 PMC8416731 34512170

[116] Thomas D Radhakrishnan P. Tumor-stromal crosstalk in pancreatic cancer and tissue fibrosis. Mol Cancer (2019) 18(1):14. doi: 10.1186/s12943-018-0927-5 30665410 PMC6341551

[117] Perera C Xu Z Mekapogu AR Hosen SMZ Pothula S Cohen-Hyams T. Effects of pancreatic stellate cell-and cancer cell-derived exosomes on islet cell functions: implications for pancreatic cancer-related diabetes. J Gastroenterol Hepatol (Australia) (2020) 35(SUPPL 1):23. doi: 10.1111/jgh.15268

[118] Garcia-Contreras M Brooks RW Boccuzzi L Robbins PD Ricordi C. Exosomes as biomarkers and therapeutic tools for type 1 diabetes mellitus. Eur Rev Med Pharmacol Sci (2017) 21(12):2940–56. 28682421

[119] Liu J Sun X Zhang FL Jin H Yan XL Huang S. Clinical potential of extracellular vesicles in type 2 diabetes. Front Endocrinol (Lausanne) (2020) 11:596811. doi: 10.3389/fendo.2020.596811 33551993 PMC7859486

[120] Sun Y Tao Q Wu X Zhang L Liu Q Wang L. The utility of exosomes in diagnosis and therapy of diabetes mellitus and associated complications. Front Endocrinol (Lausanne) (2021) 12:756581. doi: 10.3389/fendo.2021.756581 34764939 PMC8576340

[121] Hu W Song X Yu H Sun J Zhao Y. Therapeutic potentials of extracellular vesicles for the treatment of diabetes and diabetic complications. Int J Mol Sci (2020) 21(14):5163. doi: 10.3390/ijms21145163 32708290 PMC7404127

[122] Ashcroft BA de Sonneville J Yuana Y Osanto S Bertina R ME K. Determination of the size distribution of blood microparticles directly in plasma using atomic force microscopy and microfluidics. BioMed Microdevices (2012) 14(4):641–9. doi: 10.1007/s10544-012-9642-y PMC3388260 22391880

[123] Coumans FAW Brisson AR Buzas EI Dignat-George F Drees EEE El-Andaloussi S. Methodological guidelines to study extracellular vesicles. Circ Res (2017) 120(10):1632–48. doi: 10.1161/CIRCRESAHA.117.309417 28495994

[124] Greening DW Xu R Ji H Tauro BJ Simpson RJ. A protocol for exosome isolation and characterization: evaluation of ultracentrifugation, density-gradient separation, and immunoaffinity capture methods. Methods Mol Biol (2015) 1295:179–209. doi: 10.1007/978-1-4939-2550-6_15 25820723

[125] Konoshenko MY Lekchnov EA Vlassov AV Laktionov PP. Isolation of extracellular vesicles: general methodologies and latest trends. BioMed Res Int (2018) 2018:8545347. doi: 10.1155/2018/8545347 29662902 PMC5831698

[126] Livshits MA Khomyakova E Evtushenko EG Lazarev VN Kulemin NA Semina SE. Isolation of exosomes by differential centrifugation: theoretical analysis of a commonly used protocol. Sci Rep (2015) 5:17319. doi: 10.1038/srep17319 26616523 PMC4663484

[127] Zhang Y Bi J Huang J Tang Y Du S Li P. Exosome: a review of its classification, isolation techniques, storage, diagnostic and targeted therapy applications. Int J nanomed (2020) 15:6917–34. doi: 10.2147/ijn.S264498 PMC7519827 33061359

[128] Carnino JM Lee H Jin Y. Isolation and characterization of extracellular vesicles from broncho-alveolar lavage fluid: a review and comparison of different methods. Respir Res (2019) 20(1):240. doi: 10.1186/s12931-019-1210-z 31666080 PMC6822481

[129] Brisson AR Tan S Linares R Gounou C Arraud N. Extracellular vesicles from activated platelets: a semiquantitative cryo-electron microscopy and immuno-gold labeling study. Platelets (2017) 28(3):263–71. doi: 10.1080/09537104.2016.1268255 28102751

[130] Thery C Amigorena S Raposo G Clayton A. Isolation and characterization of exosomes from cell culture supernatants and biological fluids. Curr Protoc Cell Biol (2006) 30(1):3–22. doi: 10.1002/0471143030.cb0322s30 18228490

[131] Buschmann D Mussack V Byrd JB. Separation, characterization, and standardization of extracellular vesicles for drug delivery applications. Advanced Drug delivery Rev (2021) 174:348–68. doi: 10.1016/j.addr.2021.04.027 PMC8217305 33964356

[132] Islam MK Syed P Lehtinen L Leivo J Gidwani K Wittfooth S. A nanoparticle-based approach for the detection of extracellular vesicles. Sci Rep (2019) 9(1):10038. doi: 10.1038/s41598-019-46395-2 31296879 PMC6624270

[133] Paganini C Hettich B Kopp MRG Eordogh A Capasso Palmiero U Adamo G. Rapid characterization and quantification of extracellular vesicles by fluorescence-based microfluidic diffusion sizing. Adv Healthc Mater (2022) 11(5):e2100021. doi: 10.1002/adhm.202100021 34109753 PMC11469030

[134] Krishnan P Syed F Jiyun Kang N Mirmira RG Evans-Molina C. Profiling of rnas from human islet-derived exosomes in a model of type 1 diabetes. Int J Mol Sci (2019) 20(23):5903. doi: 10.3390/ijms20235903 31775218 PMC6928620

[135] Fu Q Jiang H Wang Z Wang X Chen H Shen Z. Injury factors alter mirnas profiles of exosomes derived from islets and circulation. Aging (Albany NY) (2018) 10(12):3986–99. doi: 10.18632/aging.101689 PMC6326691 30552311

[136] Pang H Luo S Xiao Y Xia Y Li X Huang G. Emerging roles of exosomes in T1dm. Front Immunol (2020) 11:593348. doi: 10.3389/fimmu.2020.593348 33324409 PMC7725901

[137] Negi S Rutman AK Paraskevas S. Extracellular vesicles in type 1 diabetes: messengers and regulators. Curr Diabetes Rep (2019) 19(9):69. doi: 10.1007/s11892-019-1193-7 31367976

[138] Gao F Chiu SM Motan DA Zhang Z Chen L Ji HL. Mesenchymal stem cells and immunomodulation: current status and future prospects. Cell Death Dis (2016) 7(1):e2062. doi: 10.1038/cddis.2015.327 26794657 PMC4816164

[139] Abdi R Fiorina P Adra CN Atkinson M Sayegh MH. Immunomodulation by mesenchymal stem cells: a potential therapeutic strategy for type 1 diabetes. Diabetes (2008) 57(7):1759–67. doi: 10.2337/db08-0180 PMC2453631 18586907

[140] Campbell JE Drucker DJ. Islet α cells and glucagon–critical regulators of energy homeostasis. Nat Rev Endocrinol (2015) 11(6):329–38. doi: 10.1038/nrendo.2015.51 25850661

[141] Mathis D Vence L Benoist C. Beta-cell death during progression to diabetes. Nature (2001) 414(6865):792–8. doi: 10.1038/414792a 11742411

[142] Perreault L Skyler JS Rosenstock J. Novel therapies with precision mechanisms for type 2 diabetes mellitus. Nat Rev Endocrinol (2021) 17(6):364–77. doi: 10.1038/s41574-021-00489-y 33948015

[143] Sun Y Mao Q Shen C Wang C Jia W. Exosomes from beta-cells alleviated hyperglycemia and enhanced angiogenesis in islets of streptozotocin-induced diabetic mice. Diabetes Metab Syndrome Obesity: Targets Ther (2019) 12:2053–64. doi: 10.2147/DMSO.S213400 PMC6790122 31632115

[144] Rickels MR Robertson RP. Pancreatic islet transplantation in humans: recent progress and future directions. Endocr Rev (2019) 40(2):631–68. doi: 10.1210/er.2018-00154 PMC6424003 30541144

[145] de Kort H de Koning EJ Rabelink TJ Bruijn JA Bajema IM. Islet transplantation in type 1 diabetes. BMJ (Clinical Res ed) (2011) 342:d217. doi: 10.1136/bmj.d217 21257658

[146] Huang X Moore DJ Ketchum RJ Nunemaker CS Kovatchev B McCall AL. Resolving the conundrum of islet transplantation by linking metabolic dysregulation, inflammation, and immune regulation. Endocr Rev (2008) 29(5):603–30. doi: 10.1210/er.2008-0006 PMC2819735 18664617

[147] Zheng X Zheng X Wang X Ma Z Gupta Sunkari V Botusan I. Acute hypoxia induces apoptosis of pancreatic β-cell by activation of the unfolded protein response and upregulation of chop. Cell Death Dis (2012) 3(6):e322. doi: 10.1038/cddis.2012.66 22695615 PMC3388238

[148] Röder PV Wu B Liu Y Han W. Pancreatic regulation of glucose homeostasis. Exp Mol Med (2016) 48(3):e219. doi: 10.1038/emm.2016.6 26964835 PMC4892884

[149] Wiklander OPB Brennan M Lötvall J Breakefield XO El Andaloussi S. Advances in therapeutic applications of extracellular vesicles. Sci Transl Med (2019) 11(492):eaav8521. doi: 10.1126/scitranslmed.aav8521 31092696 PMC7104415

[150] Lund A Bagger JI Christensen M Knop FK Vilsboll T. Glucagon and type 2 diabetes: the return of the alpha cell. Curr Diabetes Rep (2014) 14(12):555. doi: 10.1007/s11892-014-0555-4 25344790

[151] Rorsman P Huising MO. The somatostatin-secreting pancreatic Δ-cell in health and disease. Nat Rev Endocrinol (2018) 14(7):404–14. doi: 10.1038/s41574-018-0020-6 PMC5997567 29773871

[152] Soria B Andreu E Berna G Fuentes E Gil A Leon-Quinto T. Engineering pancreatic islets. Pflugers Arch (2000) 440(1):1–18. doi: 10.1007/s004240000251 10863992

[153] Schwartz MW Seeley RJ Tschöp MH Woods SC Morton GJ Myers MG. Cooperation between brain and islet in glucose homeostasis and diabetes. Nature (2013) 503(7474):59–66. doi: 10.1038/nature12709 24201279 PMC3983910

[154] Shi Y Park KS Kim SH Yu J Zhao K Yu L. Iggs from patients with amyotrophic lateral sclerosis and diabetes target Ca(V)α(2)Δ1 subunits impairing islet cell function and survival. Proc Natl Acad Sci United States America (2019) 116(52):26816–22. doi: 10.1073/pnas.1911956116 PMC6936400 31826954

[155] Baloni P Funk CC Readhead B Price ND. Systems modeling of metabolic dysregulation in neurodegenerative diseases. Curr Opin Pharmacol (2021) 60:59–65. doi: 10.1016/j.coph.2021.06.012 34352486 PMC8511060

[156] Liu S Wu X Chandra S Lyon C Ning B Jiang L. Extracellular vesicles: emerging tools as therapeutic agent carriers. Acta Pharm Sin B (2022) 12(10):3822–42. doi: 10.1016/j.apsb.2022.05.002 PMC9532556 36213541

[157] Yerneni SS Lathwal S Shrestha P Shirwan H Matyjaszewski K Weiss L. Rapid on-demand extracellular vesicle augmentation with versatile oligonucleotide tethers. ACS Nano (2019) 13(9):10555–65. doi: 10.1021/acsnano.9b04651 PMC6800810 31436946

[158] Rayamajhi S Nguyen TDT Marasini R Aryal S. Macrophage-derived exosome-mimetic hybrid vesicles for tumor targeted drug delivery. Acta Biomater (2019) 94:482–94. doi: 10.1016/j.actbio.2019.05.054 31129363

[159] S ELA Mäger I Breakefield XO Wood MJ. Extracellular vesicles: biology and emerging therapeutic opportunities. Nat Rev Drug Discovery (2013) 12(5):347–57. doi: 10.1038/nrd3978 23584393

[160] Batrakova EV Kim MS. Using exosomes, naturally-equipped nanocarriers, for drug delivery. J Controlled release (2015) 219:396–405. doi: 10.1016/j.jconrel.2015.07.030 PMC4656109 26241750

